# A283 HETEROGENEITY OF TREATMENT RESPONSE TO BETA-BLOCKERS IN THE TREATMENT OF PORTAL HYPERTENSION RELATED TO CIRRHOSIS

**DOI:** 10.1093/jcag/gwad061.283

**Published:** 2024-02-14

**Authors:** M Alsaeid, J Abraldes

**Affiliations:** University of Alberta, Edmonton, AB, Canada; University of Alberta, Edmonton, AB, Canada

## Abstract

**Background:**

Non-selective beta-blockers (BB) improve clinical outcomes in patients with cirrhosis and portal hypertension. However, it has been suggested that only a proportion of patients treated with BB benefit from them. Indeed, patients who achieve a ampersand:003E20% reduction in Hepatic Venous Pressure Gradient (HVPG) with BB have an excellent prognosis, but only 30-50% achieve such response. This has been suggested as a reason for not using BB where no HVPG measurements are available.

**Aims:**

In this study we aimed to quantify how heterogeneous is the response to BB in patients with cirrhosis, by analyzing trials in which the effects of BB on HVPG were compared with those of placebo.

**Methods:**

For assessing the potential heterogeneity of treatment response to BB we conducted a meta-analysis of differences in variance between trial arms. The degree of heterogeneity of HVPG response to BB was quantified with the pooled variability ratio (VR) (SD of the HVPG at the end of the trial in the BB group divided by that in the placebo group).

**Results:**

Our systematic search yielded 18 studies. Figure 1 shows a forest plot with the meta-analysis of the variability ratios (VR) in final HVPG. Pooled VR was 0.99 (95% CI 0.87-1.14). This indicates that there was no evidence for a higher average variability in the final HVPG in the beta-blocker treatment groups as compared to placebo groups, and hence there was no evidence to support that patients with cirrhosis exhibit a heterogeneous response to beta-blockers (i.e. there is no evidence to support that some patients responded to beta-blockers and others did not).

**Conclusions:**

In conclusion, the analysis of RCTs comparing the HVPG response of beta-blockers with placebo in patients with cirrhosis does not suggest a heterogeneous hemodynamic response to beta-blockers. This, together with the fact that in most RCTs demonstrating the clinical benefits of beta-blockers treatment was not adjusted based on HVPG response, further supports the concept that there is no need to perform portal pressure measurements to guide treatment with beta-blockers.

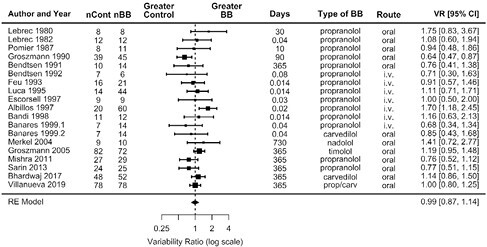

**Funding Agencies:**

None

